# Rituximab-Induced Bronchiolitis Obliterans Organizing Pneumonia

**DOI:** 10.1155/2012/680431

**Published:** 2012-06-19

**Authors:** Ahmet B. Ergin, Nancy Fong, Hamed A. Daw

**Affiliations:** ^1^Department of Medicine, Fairview Hospital, Cleveland, OH 44111, USA; ^2^Department of Pathology, Cleveland Clinic Cancer Center, Fairview Hospital, Cleveland, OH 44111, USA; ^3^Moll Pavilion, Fairview Hospital, Cleveland, OH 44111, USA

## Abstract

Rituximab-induced lung disease (R-ILD) is a rare entity that should be considered in patients treated with rituximab who present with dyspnea, fever, and cough, but no clear evidence of infection. A variety of pathologic findings have been described in this setting. Bronchiolitis obliterans organizing pneumonia (BOOP) is the most common clinicopathologic diagnosis, followed by interstitial pneumonitis, acute respiratory distress syndrome (ARDS), and hypersensitivity pneumonitis. Prompt diagnosis and treatment with corticosteroids are essential as discussed by Wagner et al. (2007). Here we present a case of an 82-year-old man who was treated with rituximab for recurrent marginal zone lymphoma. After the first infusion of rituximab, he reported fever, chills, and dyspnea. On computed tomography imaging, he was found to have bilateral patchy infiltrates, consistent with BOOP on biopsy. In our patient, BOOP was caused by single-agent rituximab, in the first week after the first infusion of rituximab. We reviewed the relevant literature to clarify the different presentations and characteristics of R-ILD and raise awareness of this relatively overlooked entity.

## 1. Introduction

Rituximab, a mouse/human chimeric anti-CD20 antibody human monoclonal antibody has been effectively used to treat lymphoma since 1997. It has also been used for immune thrombocytopenic purpura, systemic lupus erythematous, rheumatoid arthritis, and autoimmune hemolytic anemia. Rituximab has been associated with infusion-related self-limited symptoms including fever, chills, and rigor [1]. Recently more severe lung pathologies were described. We prefer rituximab-induced lung disease (R-ILD) for this group of complications rather than rituximab-induced interstitial lung disease due to the variety of pathologic diagnoses seen in this setting. Here, we report a case of BOOP occurring in the first week of rituximab treatment and review the relevant literature.

## 2. Case Report

**Figure 1:**
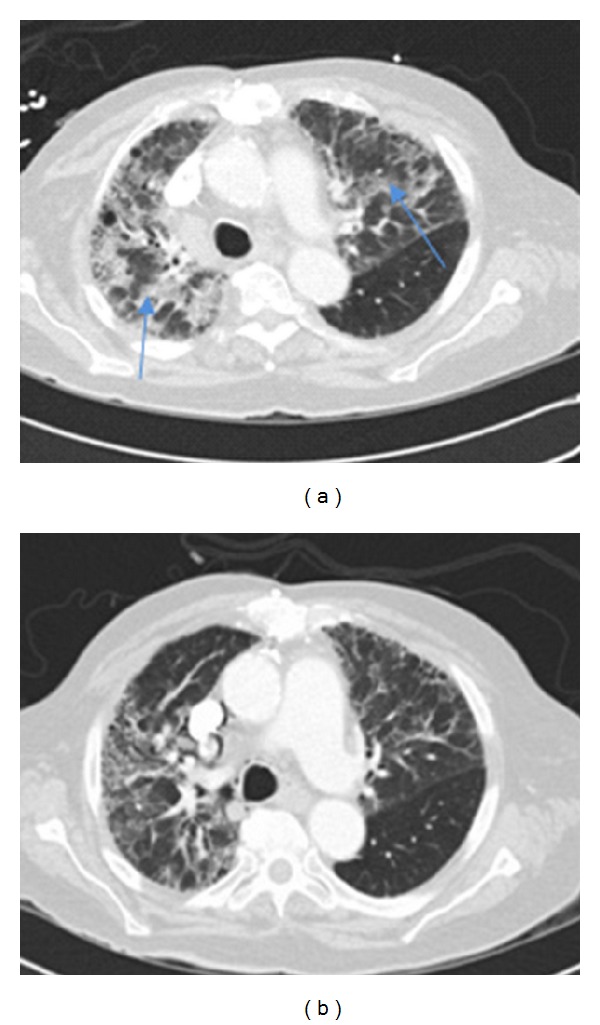
Computed tomography (CT) of the chest at the initial presentation of patient on the fifth day following rituximab treatment (a) and 20 days after initiation of steroid treatment (b). Arrows show bilateral pulmonary patchy infiltrates.

The patient was an 82-year-old male with recurrent nodal marginal zone B-cell stage 4 lymphoma, mostly involving abdominal lymph nodes. His past medical history included IgG kappa monoclonal gammopathy, congestive heart failure, sick sinus syndrome, and hypertension. The patient presented to the hospital complaining of chills, fever, and dyspnea for two days, 4 days after receiving his first infusion of rituximab therapy (375 mg/m^2^) with a premedication including acetaminophen and diphenhydramine but not steroids. No history of recent upper respiratory infection, chest pain, acute blood loss, or new medications was reported. The patient was admitted to the hospital and started on broad-spectrum antibiotics. His routine blood counts, liver function test, renal function test, and D-dimer were normal. His shortness of breath progressively worsened over the course of 3 days. The patient developed hypoxic respiratory failure. On physical examination, he was tachypneic and tachycardic. Lung auscultation revealed bibasilar inspiratory crackles. Oxygen saturation was 90% on nonrebreather mask and arterial blood gas revealed a PO_2_ of 54 mmHg on 100% FiO_2_. CT of the chest showed bilateral diffuse patchy infiltrates involving 3/4 of the lung parenchyma (Figure 1). The patient had a previous PET CT one month ago which showed mild bilateral fibrous changes in the periphery of the lungs.

**Figure 2:**
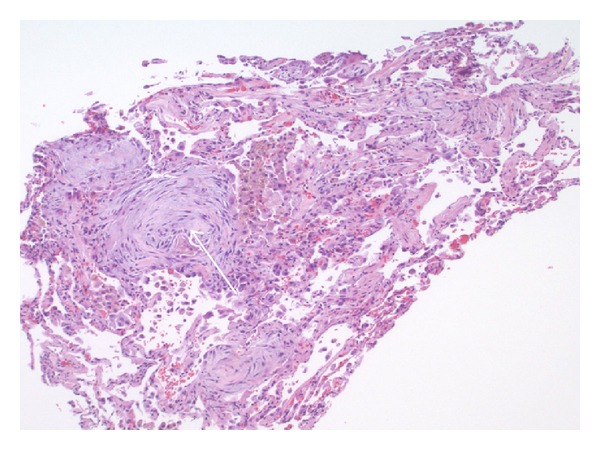
Transbronchial biopsy of the lung. Arrows point at myxoid fibroblastic plugs of bronchiolitis obliterans organizing pneumonia (BOOP).

The patient was intubated and placed on mechanical ventilation. A two-dimensional echocardiogram (2D-Echo) showed left ventricular ejection fraction (EF) of 55%, stage 1-diastolic function and no valvular disease. BNP level was 95 pg/mL. Bronchoscopy with bronchoalveolar lavage and transbronchial biopsies was performed. Bacterial, viral, and fungal cultures were negative. BAL was negative for Pneumocystis jirovecii and malignant cells. Biopsy showed pulmonary parenchyma with patchy fibroblastic proliferation, suggestive of BOOP with no malignant or atypical cells (Figure 2).

Antibiotics were discontinued and the patient was extubated, but continued to require high oxygen flow. Methylprednisolone 40 mg IV every 8 hours was started with a gradual improvement in his oxygen saturation over the next few days. Follow-up CT scan of the chest two weeks after starting steroids showed improvement in the bilateral pulmonary infiltrates. He was switched to oral prednisone at a dose 60 mg daily with a weaning plan over the next few months. After discharge to home, he no longer required oxygen.

## 3. Discussion

Two aspects are remarkable in this case report. First, BOOP presentation was early in the first week following rituximab treatment, which has not been reported before. Second, this is one of the initial reports of BOOP following single-agent rituximab treatment.

Rituximab was approved by the FDA in 1997 for lymphoma treatment. Patients are given one to six infusions at intervals depending on the type of lymphoma. Rituximab is given as a single agent or in combination regimens. It is overall a well-tolerated drug [2–5], with lung toxicity rate of less than 0.03% among 540,000 patients [2].

Notwithstanding, many life-threatening pulmonary side effects were reported [1, 6–13]. A prospective Korean study described 107 patients with non-Hodgkin lymphoma treated with a rituximab-containing regimen. Among these patients, 9 (8%) developed interstitial pneumonitis during rituximab therapy [12], suggesting a higher incidence of R-ILD than previously considered. Many factors may account for the limited number of R-ILD reported cases. The first factor is reporting bias due to poor outcomes; the second is the failure to recognize this complication by attributing symptoms to infections or to the underlying disease; and the third is the common use of corticosteroids for suspected reactive airway disease which may treat R-ILD in some patients [1].

Although presentation and clinical features are very similar, there is some variation regarding pathologic descriptions of R-ILD. Among reported cases in which pathology reports were available, the predominant finding was BOOP [8, 10, 14–19]. Interstitial pneumonia/pneumonitis with or without interstitial fibrosis was the second most common diagnosis [1, 10, 20–22]. Five ARDS cases were identified as infusion reactions to rituximab [23]. Hypersensitivity pneumonitis was reported in 3 case reports [15, 19, 21] which were also biopsy proven. In 2 cases, R-ILD was complicated by alveolar hemorrhage [15, 21].

R-ILD is a diagnosis of exclusion. Differential diagnoses includes lymphoma progression, infection, cardiogenic edema, radiation pneumonitis, pulmonary hemorrhage, and allergies. There is no clear consensus in regards to criteria for rituximab causality. Our criteria were the following.

### 3.1. Clinical Manifestation

Clinical findings of R-ILD consist of dyspnea (85%), fever (62%), and cough (43%) [23]. High-resolution CT of the chest demonstrates diffuse interstitial pattern (ground glass opacities) (34%), focal alveolar pattern (54%), and diffuse alveolar pattern (8.5%) [23]. The predominant abnormalities on pulmonary function test when performed, associates a restrictive pattern with a reduction in the diffusion capacity of CO (DLCO) [24–26]. Additionally, a bronchoscopy with bronchoalveolar lavage is required to rule out an infectious aetiology while biopsy can demonstrate interstitial fibrosis or alveolitis. We did not perform a pulmonary function test due to the rapid deterioration of our patient.

### 3.2. Rechallenge

Rechallenge has its own limitations given the possibility of fatal outcome. In a study with Lioté et al. [23], rechallenge, either intentional or unintentional, gave positive results in 4 patients with rituximab alone [10, 25, 27, 28] and in eight patients with rituximab combination therapy [10, 12, 15, 20, 26, 28]. Rechallenge gave negative results in 3 patients who also took concomitant steroid treatment [23]. 

### 3.3. Time to Onset

Lioté et al. [23] reported a mean time of symptom onset of 3 months with a peak after the fourth cycle of treatment. There was a significant variance in timing, even with similar pathologic findings. Delay from the last rituximab infusion to the onset of respiratory manifestations was about 15 days in their review. Our patient reported symptoms on the 5th day after rituximab treatment.

### 3.4. Exclusion of Other Medications

Rituximab has been given as a single agent in 7 patients with reported R-ILD [8, 18, 19, 24, 25, 27]. In other cases, rituximab was given as part of a chemotherapy regimen, most often combined with CHOP (cyclophosphamide, vincristine, doxorubicin, and prednisone). Cyclophosphamide and bleomycin are known to cause early onset pneumonitis. Among 107 patients treated with R-CHOP regimen, 9 patients developed interstitial pneumonitis when compared to none of the 66 patients treated with CHOP alone [12].

Pathogenesis of R-ILD is largely unknown [1]. Rituximab acts by binding CD20+ B cells. Toxicity and efficacy are related to events after binding, which include B-cell signaling, complement activation, direct apoptosis, and antibody-dependent cellular cytotoxicity [29]. Complement activation and cytokine secretion, in particular, seems to be the causative factors associated with rituximab infusion reactions [24, 29, 30]. TNF-*α* has been postulated to be the major inflammatory mediator in ILD pathogenesis by inducing chemokines, other inflammatory cytokines, and angiogenic factors [31]. Interestingly, serum levels of TNF-*α*, interferon gamma, and interleukin-4 were elevated only in the R-ILD patient where they were checked [32].

Corticosteroids are the cornerstone of treatment. We found 8 case reports in which patients with R-ILD expired despite steroid treatment [1, 7–13]. There was only one BOOP patient which was not biopsy-proven who died while on steroids [6]. On the basis of the proposed pathophysiology of the lung injury, anti-TNF-a directed therapy infliximab might have a role in severe cases and patients whose clinical condition worsens despite corticosteroids.

In conclusion, we presented a patient with clinical and pathological features of BOOP consistent with R-ILD. This is the first case of such an early presentation (in first week after the first infusion) and the second case report of BOOP after single-agent rituximab therapy. The patient responded well to steroids supporting its efficacy. Because symptoms at presentation are nonspecific, physicians should maintain a high index of suspicion to recognize this complication. Awareness of R-ILD is of utmost importance in order to prevent severe morbidity and mortality.
